# Impact of Treatment with Metformin on Adipocytokines in Patients with Polycystic Ovary Syndrome: A Meta-Analysis

**DOI:** 10.1371/journal.pone.0140565

**Published:** 2015-10-16

**Authors:** Wen Kong, Xun Niu, Tianshu Zeng, Meixia Lu, Lulu Chen

**Affiliations:** 1 Department of Endocrinology, Union Hospital, Tongji Medical College, Huazhong University of Science and Technology, Wuhan, 430022, China; 2 Department of Otolaryngology, Union Hospital, Tongji Medical College, Huazhong University of Science and Technology, Wuhan, 430022, China; 3 Department of Epidemiology and Biostatistics, and the Ministry of Education Key Laboratory of Environment and Health, School of Public Health, Tongji Medical College, Huazhong University of Science and Technology, Wuhan, Hubei, China; Medical University Innsbruck, AUSTRIA

## Abstract

**Background:**

Metformin is effective for the treatment of polycystic ovary syndrome, but conflicting results regarding its effect on adipocytokine levels (adiponectin, resistin, visfatin, and leptin) in patients with polycystic ovary syndrome receiving metformin treatment have been reported. To provide high-quality evidence about the effect of metformin treatment on adipocytokines in patients with polycystic ovary syndrome, relevant studies that assessed the levels of adipocytokines (adiponectin, resistin, visfatin, and leptin) in patients with polycystic ovary syndrome receiving treatment with metformin administration were reviewed and analyzed.

**Methods:**

A literature search was conducted in the SCI, PUBMED, EMBASE, and Elsevier databases, and personal contact was made with the authors. Standard mean differences and 95% confidence intervals were calculated and combined appropriately. To ensure synthesis of the best available evidence, sensitivity analyses were performed.

**Results:**

A total of 34 data sets were included in 4 different outcomes, involving 744 women with polycystic ovary syndrome and adipocytokine levels measured both before and after metformin administration. Metformin treatment was associated with significantly elevated serum adiponectin concentrations (standard mean differences [95% confidence interval], −0.43 [−0.75 to −0.11]) and decreased serum leptin concentrations (0.65 [0.26 to 1.04]), whereas no significant difference in resistin level (−0.01 [−0.49 to 0.45]) or visfatin level (−0.04 [−1.55 to 1.46]) was found.

**Conclusions:**

Metformin administration was associated with increased serum adiponectin concentrations and decreased serum leptin levels. Further study is needed to elucidate whether this apparent effect decreases the incidence of type 2 diabetes and other metabolic diseases in patients with polycystic ovary syndrome later in life.

## Introduction

The number of women with polycystic ovary syndrome (PCOS) has been continuously and rapidly increasing. Recent reports indicated that the prevalence of PCOS in women of reproductive age is 5–10% [1, 2]. About 50–70% of patients with PCOS have been confirmed to have insulin resistance and central obesity [3–5]. Both insulin resistance and central obesity predict the development of metabolic syndrome, which increases the risks of type 2 diabetes, hyperlipidemia, and cardiovascular disease [6–8]. Adipose tissue, especially visceral adipose tissue, is not only a fat storage depot but also an important endocrine organ, as it produces, synthesizes, and releases a number of bioactive proteins referred to as adipocytokines [9]. Recently, studies have demonstrated that adipocytokines are extensively implicated in the pathophysiological processes of metabolic disorders such as PCOS, insulin resistance, obesity, type 2 diabetes, and cardiovascular diseases [10]. Adiponectin is an adipocytokine synthesized in white adipose tissue. Its level is negatively correlated with obesity, insulin resistance, type 2 diabetes, coronary vascular diseases, and metabolic syndrome [11–13]. High adiponectin levels may reduce the risk of insulin resistance and type 2 diabetes [14]. Resistin, which contributes to insulin sensitivity, is another peptide secreted by the adipose tissue. Circulating resistin levels are increased in diet-induced and genetic forms of obesity animal models such as ob/ob and db/db mice. Serum resistin levels are decreased by using anti-diabetic drugs such as rosiglitazone [15]. Recent studies report a new adipocytokine, visfatin, also known as pre-B cell colony-enhancing factor, which is expressed in visceral lipid tissue and linked to metabolic syndrome. Studies have shown that visfatin expression is increased in individuals with abdominal obesity and type 2 diabetes [16]. Furthermore, leptin is a circulating hormone produced by adipocytes and is associated with insulin resistance in patients with PCOS [17, 18].

Metformin, a widely used insulin sensitizer, is an effective treatment of PCOS. It may improve ovulatory function in patients with PCOS, partly via weight loss or changes in body composition [19]. However, this hypothesis could not be confirmed with short-term treatment.

To understand how metformin improves the metabolic and hormonal disturbances in patients with PCOS, numerous studies have been conducted to determine the effect of metformin on adipocytokines in patients with PCOS, but their conclusions are far from uniform. Considering the large body of published evidence and amount of conflicting results, the primary purpose of the present study was to systematically review literature on the association between adipocytokines and metformin treatment in patients with PCOS in order to meta-analyze the best evidence available to provide high-quality data on the link between metformin treatment and adipocytokine levels in patients with PCOS.

## Materials and Methods

### Search strategy

We searched for English articles included in the SCI, PUBMED, EMBASE, and Elsevier databases. The following search terms were included:

1. (visfatin AND metformin) AND (PCOS OR polycystic ovary syndrome)
2. (adiponectin AND metformin) AND (PCOS OR polycystic ovary syndrome)
3. (resistin AND metformin) AND (PCOS OR polycystic ovary syndrome)
4. (leptin AND metformin) AND (PCOS OR polycystic ovary syndrome)

The computerized search was supplemented with a manual search of the bibliographies of all articles retrieved. Potentially relevant articles were assessed for inclusion based on prespecified inclusion and exclusion criteria.

### Eligibility of relevant studies

Studies that met the following criteria were included:

1. All study subjects were limited to adults with PCOS diagnosed consistently by using either the Rotterdam [20] or National Institute of Health (NIH) criteria [21].
2. The study reported adiponectin, leptin, resistin, or visfatin values obtained both before- and after metformin therapy.

Studies were excluded if they met the following criteria:

1. The information available was not adequate for data extraction.
2. Abstracts, letters to the editor, and case reports.

#### Data extraction

Information from each study was extracted independently by 2 reviewers by using a standardized data extraction form. The general characteristics, methodology (PCOS definition and adiponectin/resistin/visfatin/leptin measurement method), and outcomes (both the total adiponectin/resistin/visfatin/leptin means and SDs before and after metformin administration) of each study were recorded, where available, and double-checked. Where appropriate, the data set was completed through communication with the authors. Disagreements were resolved by consensus.

#### Statistical analysis

The meta-analysis was conducted by using STATA version 12.0 and Review manager 5.2. Standard mean differences (SMD) and 95% confidence intervals (CIs) of the adipocytokine levels were calculated for all eligible studies and combined by using appropriate fixed or random effects models. The Mantel-Haenszel analysis method was used for dichotomous variables, and the inverse variance method was used for continuous variables [22]. A *P* value < 0.05 was considered statistically significant.

Statistical heterogeneity was analyzed based on the *I* ^2^ value. A *P* value < 0.10 was considered statistically significant. An *I* ^2^ value < 25% was considered homogeneous; an *I* ^2^ value > 75%, as high heterogeneity; an *I* ^2^ value between 50% and 75%, as moderate heterogeneity; and an *I* ^2^ value between 25% and 50%, as low heterogeneity [22, 23]. If the *I* ^2^ value was >50%, the studies were believed to be moderately or highly heterogeneous, and the random effects model was used to combine the effect size. If the *I* ^2^ value was <50%, the studies were believed to be homogeneous or have low heterogeneity, and the fix effects model was used to combine the effect size [24, 25].

A sensitivity analysis was performed to evaluate the stability of the meta-analysis results. Forest plots were synthesized. Potential publication bias was investigated by using the funnel plot, Begg test, and Egger test.

A special meta-analysis was performed on the effect of metformin on body mass index (BMI) in patients with PCOS.

## Results

### Search results

**Fig 1:**
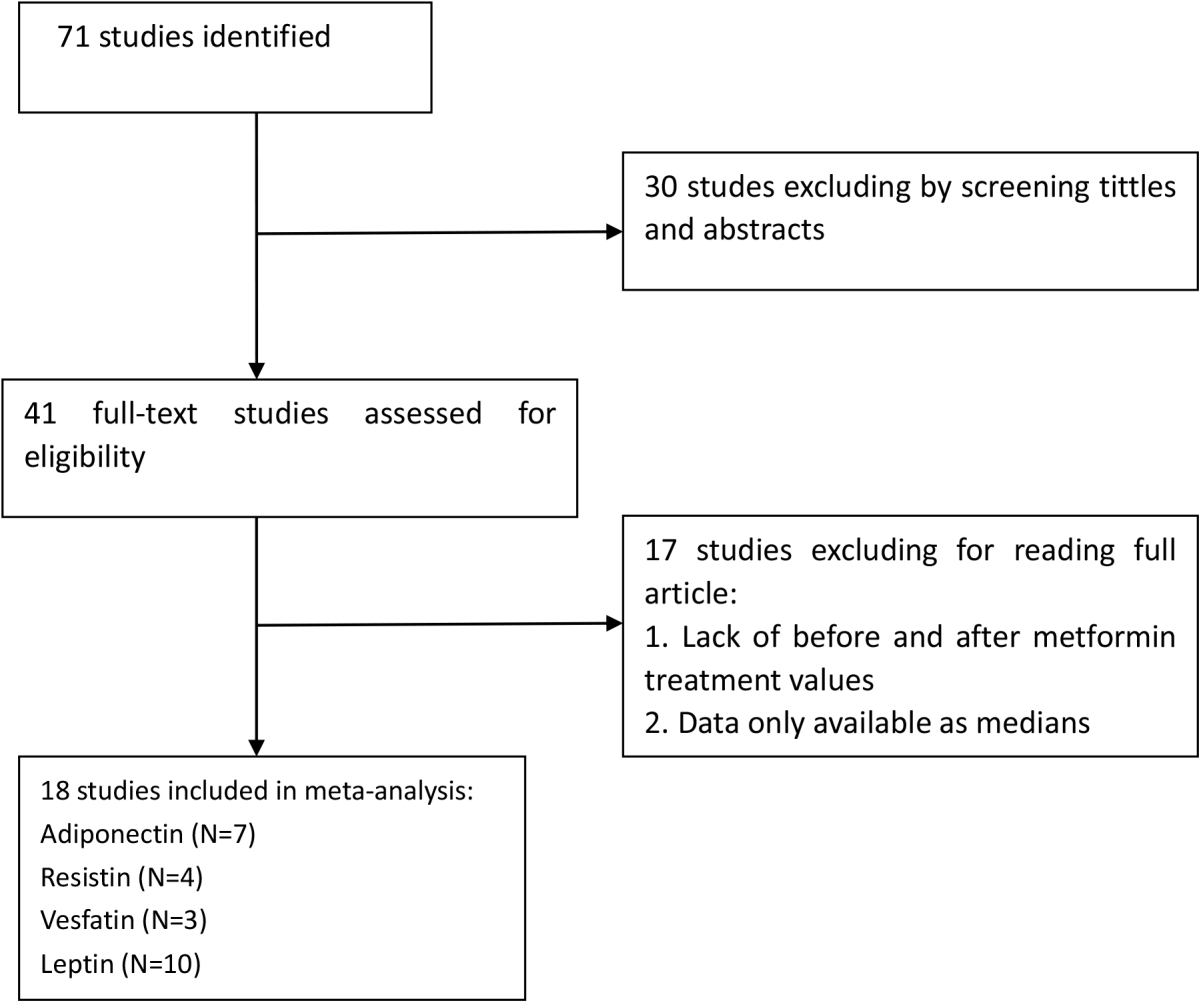
Flow diagram of the literature search.

The initial search was independently executed by 2 reviewers. The details of the steps of the literature search are shown in **Fig 1**.

### Characteristics of the Eligible Studies

Eighteen studies [26–43], with data from 744 participants, were included in this review. A total of 7 studies with 11 data sets including 280 subjects were pooled for adiponectin, 4 studies with 6 data sets including 184 subjects were pooled for resistin, 3 studies with 78 subjects were pooled for visfatin, and 10 studies with 14 data sets including 202 subjects were pooled for leptin. The characteristics of the included studies for the different adipocytokines are provided in **Tables 1–4**, respectively. All the self-control trials included were defined as level 2, and the randomized controlled trials included were defined as level 1, according to study design [44, 45].

**Table 1:** Characteristics of the 7 included studies on adiponectin.

					BMI (mean ± SD)						
Author	Year	Country	Sample size	Age (mean ± SD)	Pre-Met	Post-Met	Inclusion/exclusio n Criteria	Study design	LOE	Therapy duration (months)	Daily dose
Karen EH [27]	2008	America	15	27.7±1.3	43.3±2	42.3 ± 2	H	RCT	1	3	2000
Hossam O.H [28]	2013	Egypt	62	29.3±4.2	30.2±4.8	29.3±2.7	H	SCT	2	6	1500
Joanna J [29]	2008	Poland	29	28.24±6.27	35.32±5.07	33.49±.80	H	SCT	2	6	1000
Aleksandra [31]A	2014	Poland	32	NR	26.26±5.95	25.6±5.54	H	SCT	2	3	1000
Aleksandra [31]B	2014	Poland	32	NR	26.26±5.95	25.5±5.43	H	SCT	2	6	1000
Aleksandra [31]C	2014	Poland	16	NR	31.19±4.44	30.2±4.06	H	SCT	2	3	1000
leksandra [31]D	2014	Poland	16	NR	31.19±4.44	30.0±4.00	H	SCT	2	6	1000
Luque RM [32]A	2008	Spain	10	23±5	25.0±2.8	NR	H	RCT	1	3	850
Luque RM [32]B	2008	Spain	9	27±8	36.6±4.4	NR	H	RCT	1	3	850
Charles AS [42]	2007	Germany	35	24.7±4.8	29.3±6.5	28.6±7.2	H	RCT	1	6	1700
İlhan T [43]	2010	Turkey	24	25.21±5.99	31.69±6.52	30.7±6.65	H	SCT	2	6	1700

**Abbreviations**: SD, standard deviation; BMI, body mass index; NR, not reported; H, Have; LOE, level of evidence; SCT, self-control trials; RCT, randomized controlled trials.

**Table 2:** Characteristics of the 4 included studies on resistin.

					BMI (mean ± SD)						
Author	Year	Country	Sample size	Age (mean ± SD)	Pre-Met	Post-Met	Inclusion/exclusion criteria	Study design	LOE	Therapy duration (month)	Daily dose (mg)
Basios G [26]A	2014	Greece	31	25.7 ± 6.7	<25	NR	H	SCT	2	6	1275
Basios G [26]B	2014	Greece	31	NR	25–30	NR	H	SCT	2	6	1275
Basios G [26]C	2014	Greece	31	NR	>30	NR	H	SCT	2	6	1275
Maria M [39]	2011	Bulggaria	32	23.58±4.17	28.45± 4.38	27.45±3.73	H	SCT	2	3	1275
Charles AS [42]	2007	Germany	35	24.7±4.8	29.3 ± 6.5	28.6 ± 7.2	H	RCT	1	6	1700
İlhan T [43]	2010	Turkey	24	25.21±5.99	31.69 ± 6.52	30.7 ± 6.65	H	SCT	2	6	1700

**Abbreviations**: SD, standard deviation; BMI, body mass index; H, Have; NR, not reported; LOE, level of evidence; SCT, self-control trials; RCT, randomized controlled trials.

**Table 3:** Characteristics of the 3 included studies on visfatin.

					BMI (mean ± SD)						
Author	Year	Country	Sample size	Age (mean ± SD)	Pre-Met	Post-Met	Inclusion/exclusion criteria	Study design	LOE	Therapy duration (month)	Daily dose (mg)
Ozkaya M [40]	2008	Turkey	19	25.1 ± 3.8	27.1 ± 1.7	26.1±1.6	H	SCT	2	3	1700
Charles AS [42]	2007	Germany	35	24.7 ± 4.8	29.3 ± 6.5	28.6±7.2	H	RCT	1	6	1700
İlhan T [43]	2010	Turkey	24	25.21±5.99	31.69 ±6.52	30.7±6.65	H	SCT	2	6	1700

**Abbreviations**: SD, standard deviation; BMI, body mass index; H, have; LOE, level of evidence; SCT, self-control trials; RCT, randomized controlled trials.

**Table 4:** Characteristics of the 10 included studies on leptin.

					BMI (mean ± SD)						
Author	Year	Country	Sample size	Age (mean ± SD)	Pre-Met	Post-Met	Inclusion/exclusion criteria	Study design	LOE	Therapy duration (month)	Daily dose (mg)
Kowalska I [30]	2001	Poland	11	27.7 ± 1.3	34.9 ± 5.6	31.4±4.8	H	SCT	2	4–5	1500
Luque RM [32]A	2008	Spain	10	23 ±5	25.0 ± 2.8	NR	H	SCT	2	3	850
Luque RM [32]B	2008	Spain	9	27 ± 8	36.6 ± 4.4	NR	H	RCT	1	3	850
Maciel GA [33]	2003	Brazil	7	22.5 ± 1.9	25.3 ± 2.1	24.9±2.7	H	RCT	1	6	1500
Marciniak A [34] A	2009	Poland	23	NR	30.86 ± 5.35	30.39±6.1	H	SCT	2	3	1700
Marciniak A [34] B	2009	Poland	12	NR	20.99 ± 1.87	20.75±1.79	H	SCT	2	3	1700
Moran L J [35]	2010	Australia	29	33.5±6.7	36.1 ± 7.2	NR	H	SCT	2	6	2000
Morin PL [36] A	2003	Finland	8	28.2±1.4	22.5 ± 0.8	21.7±0.7	H	RCT	1	3	1000
Morin PL [36] B	2003	Finland	8	28.2 ±1.4	22.5 ± 0.8	22.1 ± 0.8	H	RCT	1	6	2000
Morin PL [37] A	1998	Finland	26	30 ± 6.8	31 ± 4.6	30.66± 4.7	H	SCT	2	2	1500
Morin PL [37] B	1998	Finland	12	NR	31 ± 4.6	31.7±6 5.4	H	SCT	2	4–6	1500
Ng E H [38]	2001	China	8	30.5	24.1	NR	H	RCT	2	3	1500
Maria M [39]	2011	Bulgaria	32	23.58±4.17	28.45±4.38	27.45±3.73	H	SCT	2	3	1275
Romualdi D [41]	2008	Italy	7	23.57±6.97	32.64±4.35	31.64±3.15	H	SCT	2	4	1500

**Abbreviations**: SD, standard deviation; BMI, body mass index; NR, not reported; H, have; LOE, level of evidence; SCT, self-control trials; RCT, randomized controlled trials.

### Adiponectin

#### Selection of studies

There were 23 potentially eligible studies identified. Three were excluded because they did not fulfill the standard quality requirements. Thirteen studies were excluded because only median data were available. Finally, 7 studies [27–29, 31, 32, 42, 43] were considered as having the best available evidence.

#### Main analysis

**Fig 2:**
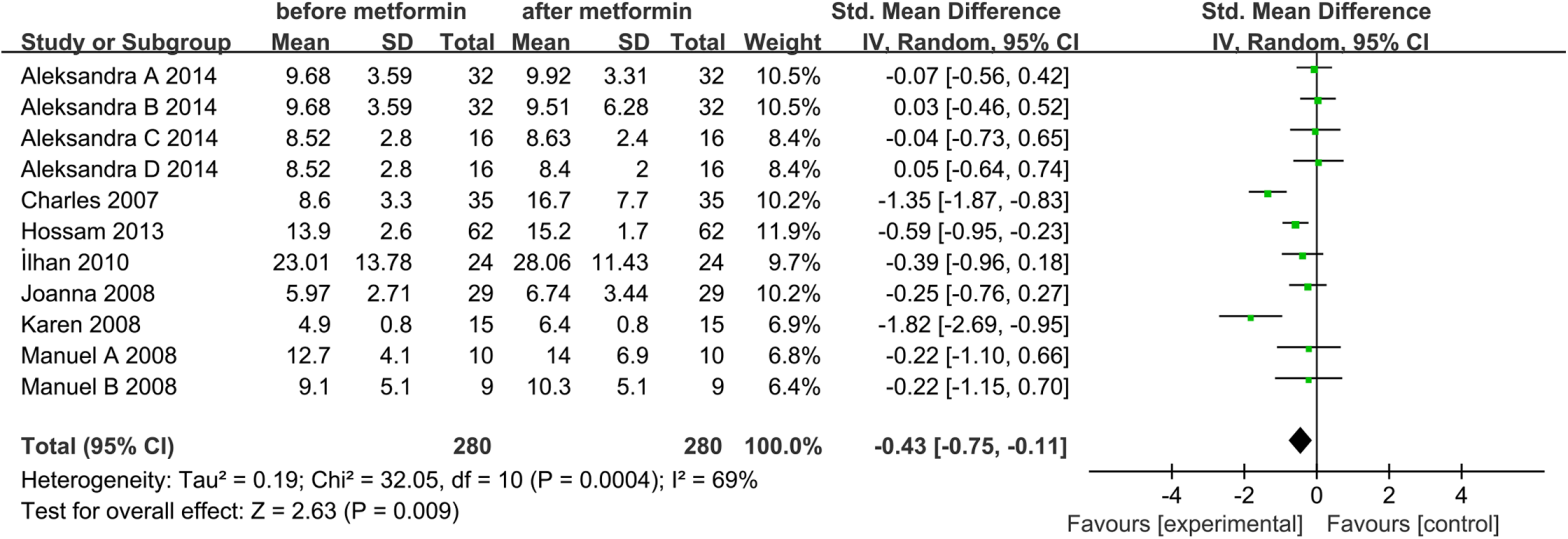
Adiponectin level. Adiponectin serum concentrations in the women with polycystic ovary syndrome before and after metformin treatment.

Seven studies, including 280 women, were eligible for the meta-analysis [27–29, 31, 32, 42, 43]. The *I* ^2^ value was 69%, indicating moderate heterogeneity among the included studies. Therefore, we used the random effects model to combine effect size. The meta-analysis revealed that metformin treatment was associated with a significant increases in serum adiponectin concentrations in the patients with PCOS, with a corresponding SMD of -0.43 (95% CI: -0.75 to -0.11, *P*<0.05) (**Fig 2**).

**Fig 3:**
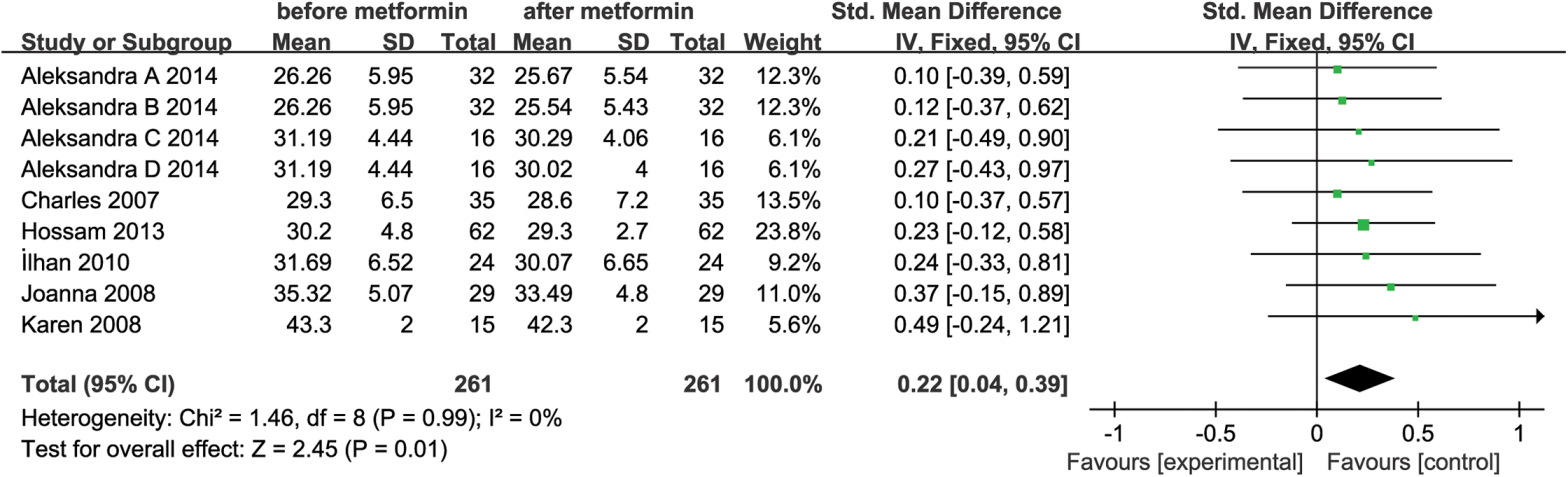
Comparison of body mass index (BMI) before and after metformin treatment in the adiponectin-related studies.

We also performed a special meta-analysis regarding the effect of metformin administration on BMI in patients with PCOS. The results showed that the total SMD for BMI was 0.22 units (95% CI, 0.04 to 0.39, P = 0.01) (**Fig 3**). The result suggests that metformin therapy was associated with a significant decrease in BMI in the women with PCOS.

#### Sensitivity Analysis

Sensitivity analysis revealed that removal of any study from the analysis did not subvert the result of the present pooled analysis (data not shown). Pooled analysis using the random effects model revealed that adiponectin levels significantly increased after metformin therapy (SMD: -0.43, 95% CI: -0.75 to -0.11, *P*<0.05). The fixed-effects model produced a similar result (SMD: -0.43, 95% CI: -0.60 to -0.26, *P*<0.05). Therefore, this pooled analysis outcome could be regarded with a higher degree of certainty.

#### Publication bias

**Fig 4:**
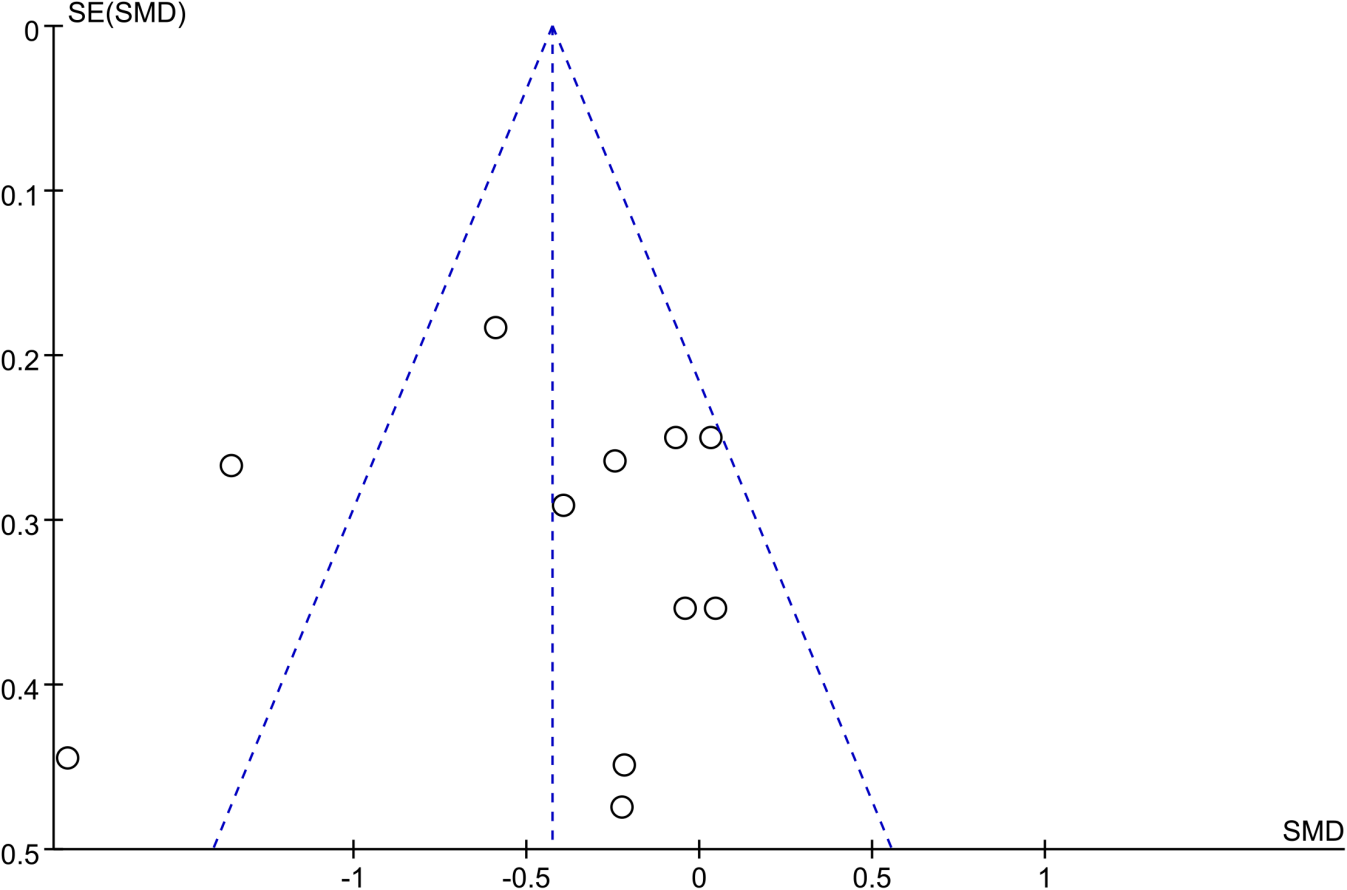
Adiponectin funnel plot. The funnel plot shows the possibility of a small publication bias. SE, standard error; SMD, standardized mean difference.

The funnel plot was not perfectly symmetrical (**Fig 4**), suggesting possible slight publication bias. However, Begg tests (*P* = 0.35) and Egger tests (*P* = 0.09) revealed no evidence to support publication bias in this pooled analysis. In addition, in the trim and fill method, none of the studies needed to be statistically corrected for funnel plot asymmetry.

### Resistin

#### Selection of studies

Seven potentially eligible studies were identified. One study was excluded, as data were not available in an extractable format. The 6 articles were then roughly screened based on their abstracts and titles, according to the inclusion/exclusion criteria. After careful discussion between the 2 reviewers, articles that fulfilled the inclusion criteria of our study were identified [26, 39, 42, 43].

#### Main analysis

**Fig 5:**
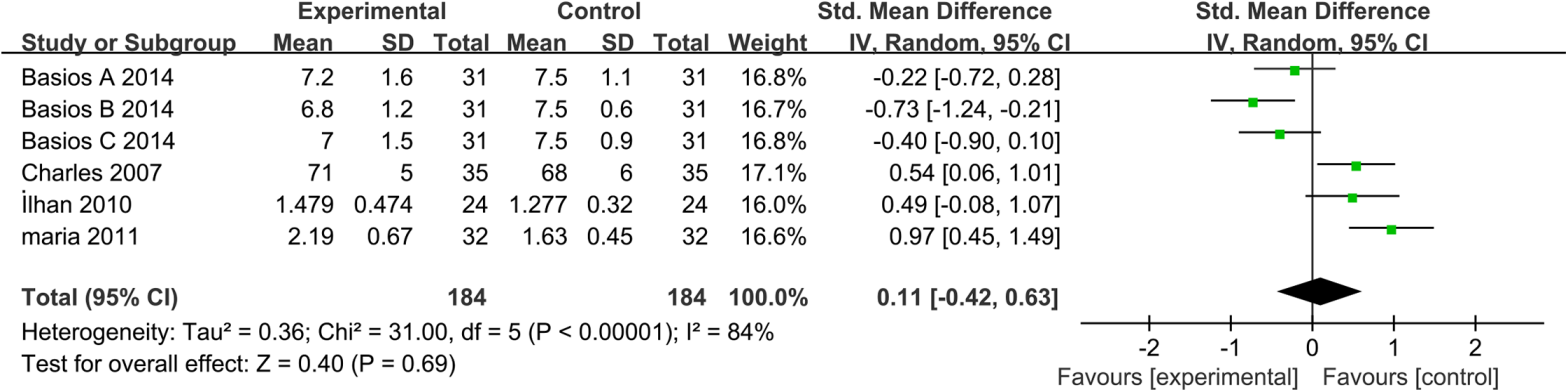
Resistin level. Resistin serum concentration in the patients with polycystic ovary syndrome before and after metformin treatment.

Four studies, involving 184 women, were eligible for the meta-analysis [26, 39, 42, 43]. An *I* ^2^ value of 84% indicated that high heterogeneity between the included studies. Therefore, the random effects model was performed to combine effect size. The meta-analysis revealed that circulating resistin concentration was not significantly changed after metformin treatment in the patients with PCOS, with a corresponding SMD of -0.01 (95% CI: -0.49 to 0.45, *P*>0.05) (**Fig 5**).

**Fig 6:**
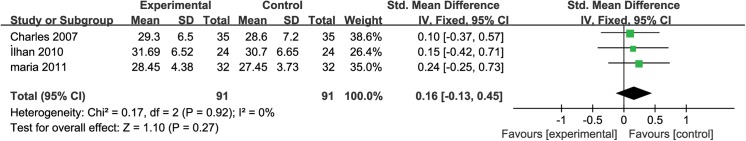
Comparison of body mass index (BMI) before and after metformin treatment in the resistin-related studies.

We also performed a special meta-analysis regarding the effect of metformin administration on BMI in the patients with PCOS. The results showed that the total SMD for BMI was 0.16 units (95% CI, -0.13 to 0.45, P = 0.27) (**Fig 6**). The result suggests that metformin therapy exerted no significant effect on BMI in the women with PCOS.

#### Sensitivity Analysis

Pooled analysis using the random effects model revealed that circulating resistin concentration was not significantly changed after metformin treatment in the patients with PCOS (SMD: -0.01, 95% CI: -0.49 to 0.45, *P*>0.05). The same conclusion was reached with the fixed-effects model (SMD: -0.10, 95% CI: -0.11 to 0.31, *P*>0.05). Meanwhile, sensitivity analysis revealed that removal of any study from the analysis did not subvert the result of the present pooled analysis (data not shown). Therefore, this pool analysis outcome could be regarded with a higher degree of certainty.

#### Publication bias

**Fig 7:**
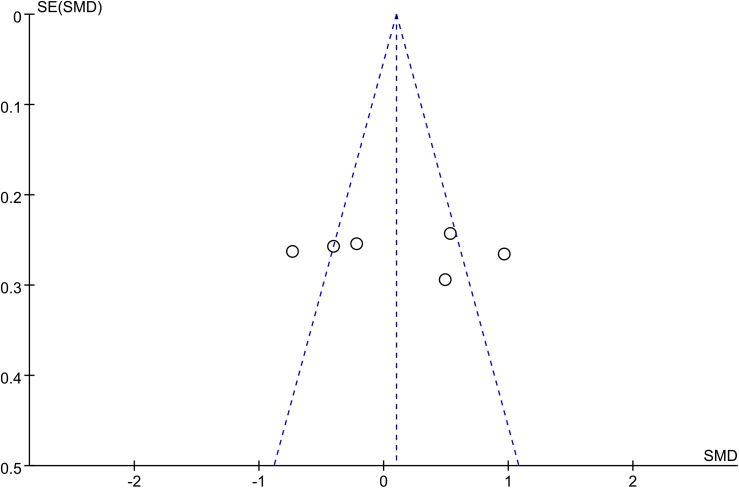
Resistin funnel plot. The funnel plot shows the possibility of a small publication bias. SE, standard error; SMD, standardized mean difference.

The funnel plot was not perfectly symmetrical (**Fig 7**). This seems to demonstrate slight publication bias. However, Begg tests (P = 0.27) and Egger tests (P = 0.13) revealed no evidence to support publication bias in this pooled analysis. In addition, in the trim and fill method, none of the studies needed to be statistically corrected for funnel plot asymmetry.

### Visfatin

#### Selection of studies

Six potentially eligible studies were identified. Three studies were excluded because they did not fulfill the standard quality requirements. The remaining 3 studies were included in the meta-analysis [40, 42, 43].

#### Main analysis

**Fig 8:**
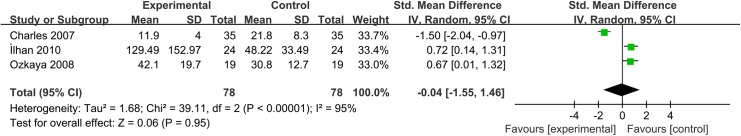
Visfatin level. Visfatin serum concentration in the women with polycystic ovary syndrome before and after metformin treatment.

Meta-analysis included 3 studies, involving 184 women [40, 42, 43]. An *I* ^2^ value of 95% indicated high heterogeneity between the included studies. Therefore, the random effects model was used to combine effect size. The results showed that metformin administration did not change serum visfatin levels in the patients with PCOS, with a corresponding SMD of -0.04 (95% CI: -1.55 to 1.46, *P*>0.05) (**Fig 8**).

**Fig 9:**
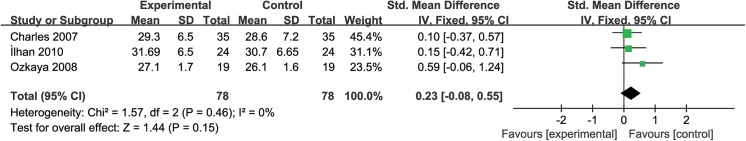
Comparison of body mass index (BMI) before and after metformin treatment in the visfatin-related studies.

We also performed a special meta-analysis regarding the effect of metformin administration on BMI in patients with PCOS. The results showed that the total SMD for BMI was 0.23 units (95% CI, -0.08 to 0.55, P = 0.15) (**Fig 9**). The result suggests that metformin therapy exerted no significant effect on BMI in the women with PCOS.

#### Sensitivity Analysis

Pooled analysis using the random effects model revealed that metformin administration did not change serum visfatin levels in the patients with PCOS (SMD: -0.04, 95% CI: -1.55 to 1.46, *P*>0.05). The fixed-effects model produced the same conclusion (SMD: -0.18, 95% CI: -0.52 to 0.15, *P*>0.05). Conversely, sensitivity analysis showed that removal of any study from the analysis could subvert the result of the present pooled analysis (data not shown). Therefore, this pooled analysis outcome may not be regarded with a higher degree of certainty.

#### Publication bias

**Fig 10:**
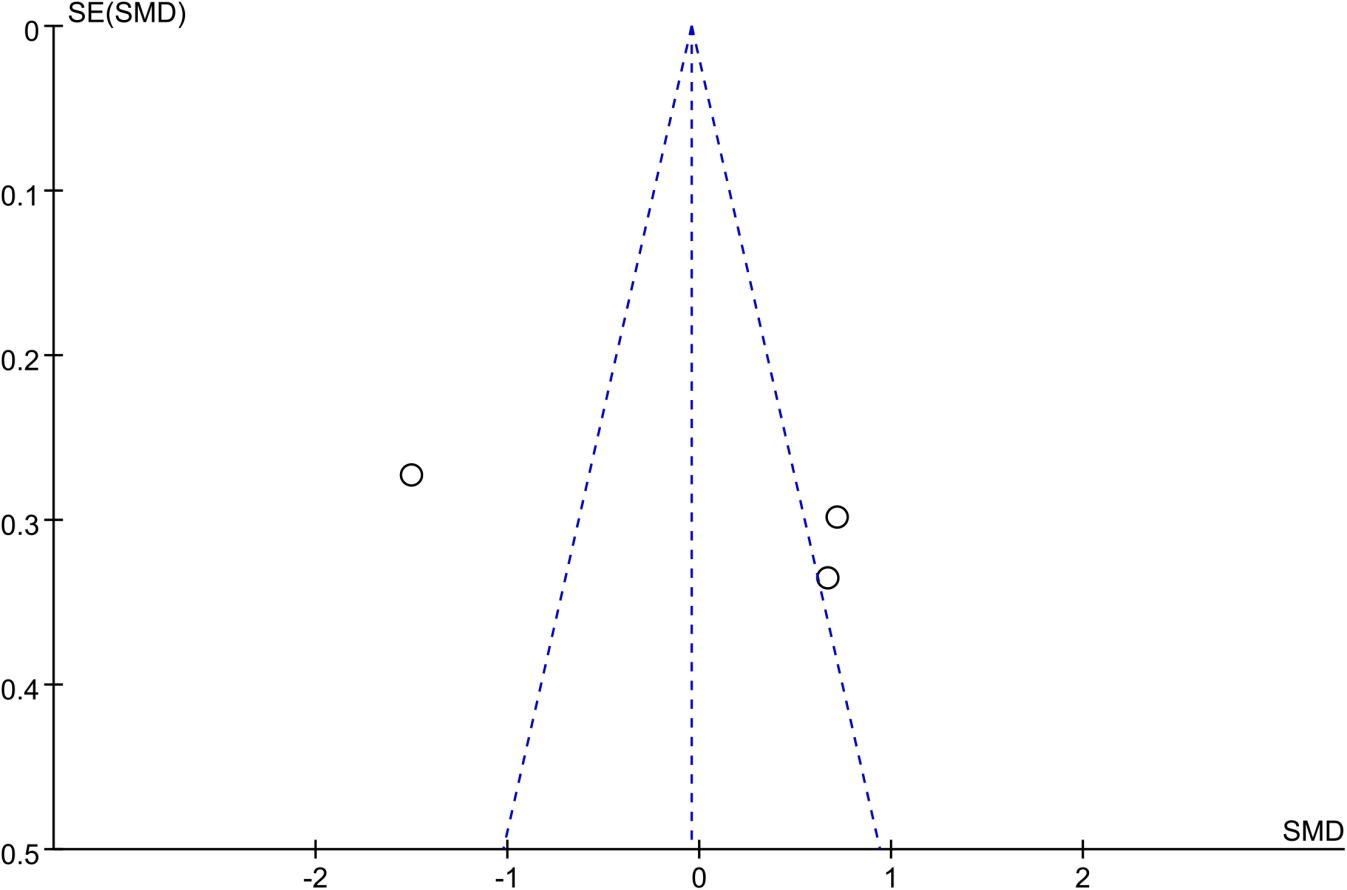
Visfatin funnel plot. The funnel plot shows the possibility of a small publication bias. SE, standard error; SMD, standardized mean difference.

The funnel plot was highly asymmetrical (**Fig 10**). This indicated a clear publication bias. To better understand the effect of metformin on serum visfatin concentration in patients with PCOS, a well-designed, large-scale, high-quality, multicenter randomized controlled trial is needed.

### Leptin

#### Selection of studies

We identified 35 potentially eligible studies. These studies were roughly screened based on their abstracts and titles, according to the inclusion/exclusion criteria. Twenty-two studies were excluded because they did not fulfill the standard quality requirements. Three studies were excluded because relevant data were not extractable, being available only as medians or in abstracts. Finally, 10 studies were considered as having the best available evidence [30, 32–39, 41].

#### Main analysis

**Fig 11:**
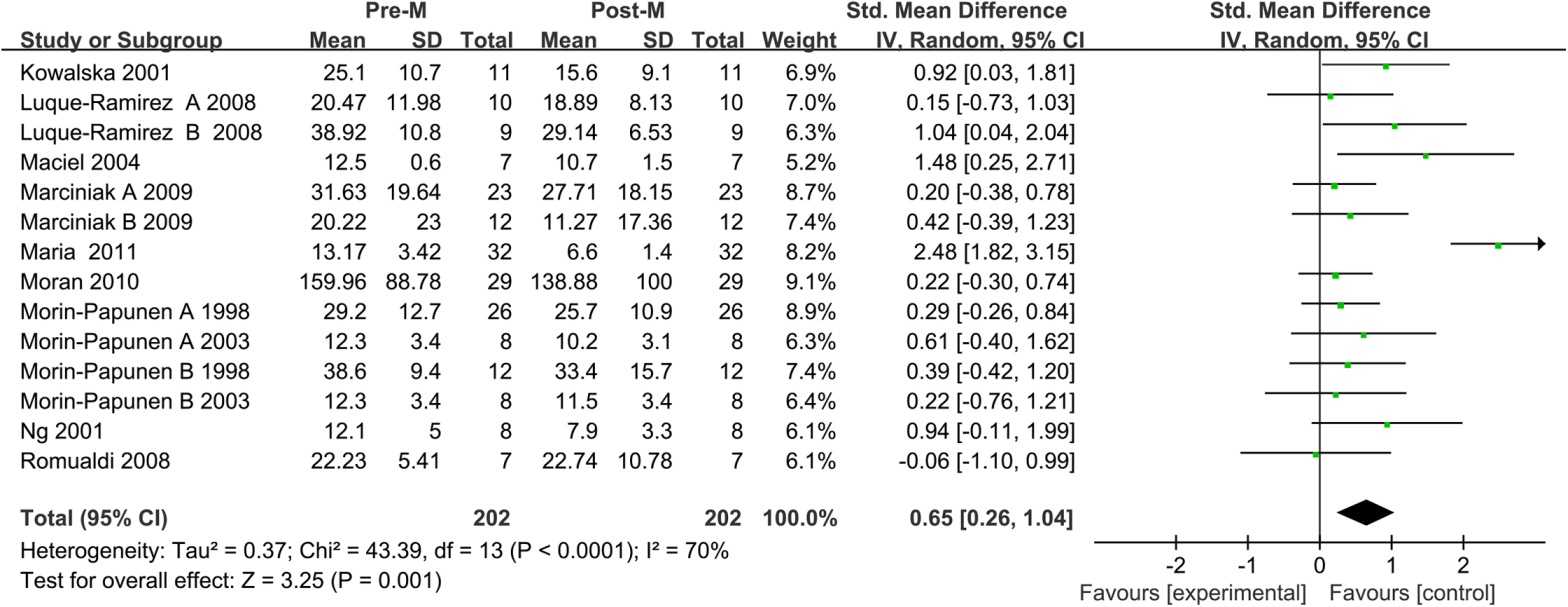
Leptin level. Leptin serum concentration in the patients with polycystic ovary syndrome before and after metformin treatment.

Meta-analysis included 10 studies, involving 202 women. An *I* ^2^ value of 70% indicated moderate heterogeneity between the included studies. Therefore, the random effects model was used to combine effect size. The meta-analysis revealed that metformin administration demonstrated an association with significantly lower leptin concentrations after treatment than before treatment in the patients with PCOS, with a corresponding SMD of 0.65 (95% CI: 0.26 to1.04, *P*<0.05) (**Fig 11**).

**Fig 12:**
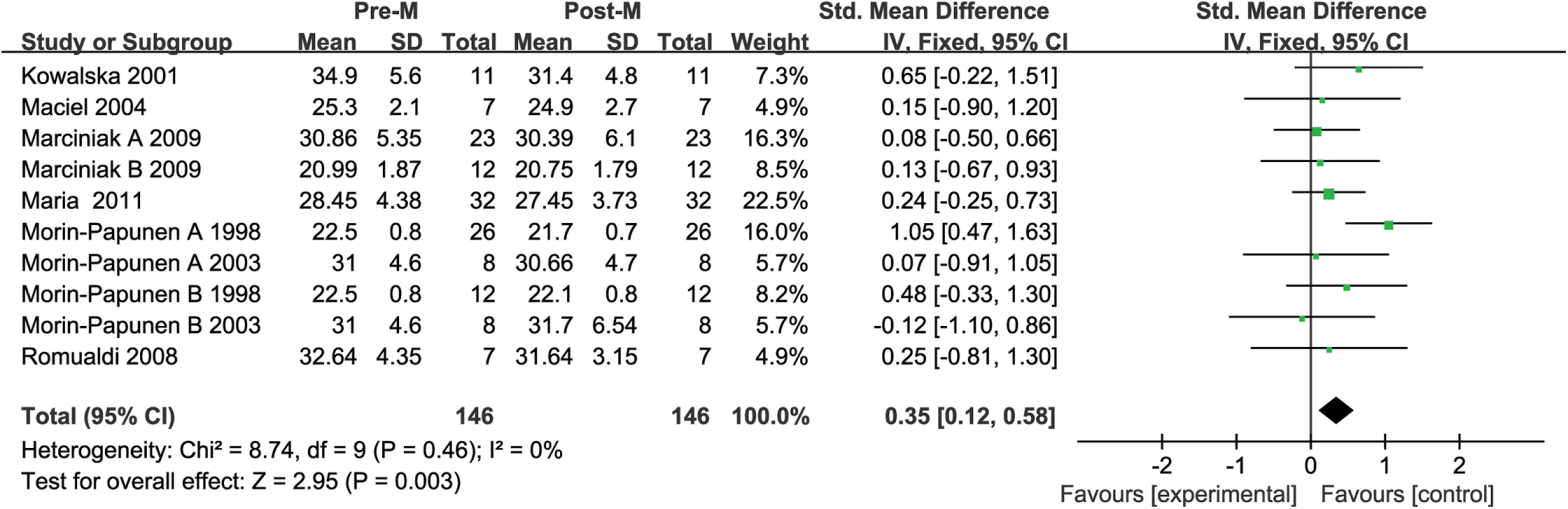
Comparison of body mass index (BMI) before and after metformin treatment in the leptin-related studies.

We also performed a special meta-analysis regarding the effect of metformin administration on BMI in patients with PCOS. The results showed that the total SMD for BMI was 0.35 units (95% CI, 0.12 to 0.58, P = 0.01) (**Fig 12**). The result suggests that metformin therapy was associated with a significant decrease in BMI in the women with PCOS.

#### Sensitivity Analysis

Sensitivity analysis showed that removal of any study from the analysis did not subvert the result of the present pooled analysis (data not shown). Pooled analysis using the random effects model revealed that leptin levels significantly increased after metformin therapy (SMD: 0.65, 95% CI: 0.26 to 1.04, *P*<0.05). The fixed-effects model produced the same result (SMD: 0.61, 95% CI: 0.40 to 0.82, *P*<0.05). Therefore, the outcome of this pool analysis could be regarded with a higher degree of certainty.

#### Publication bias

**Fig 13:**
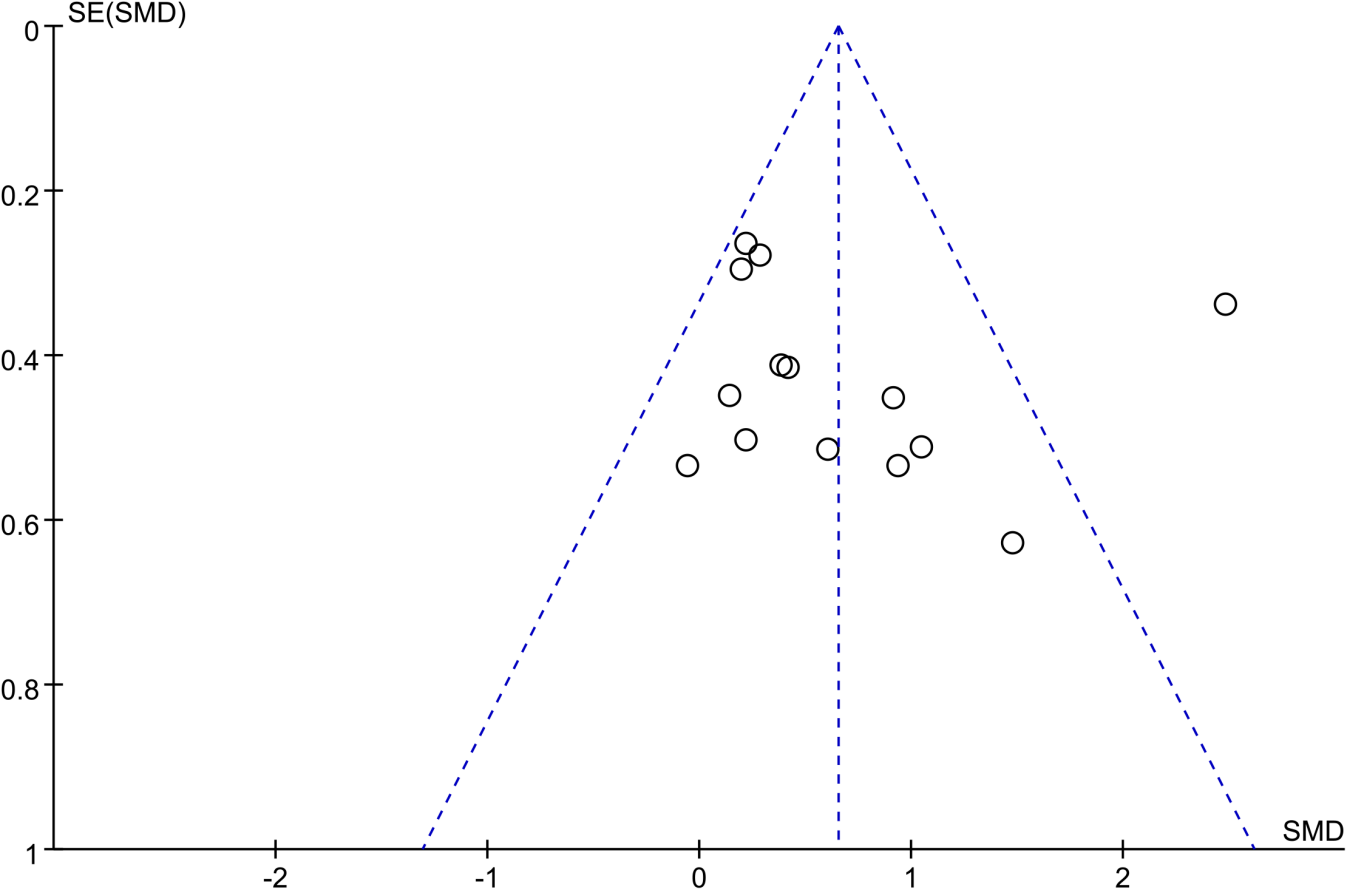
Leptin funnel plot. The funnel plot shows the possibility of a small publication bias. SE, standard error; SMD, standardized mean difference.

The funnel plot was not perfectly symmetrical (**Fig 13**). This seemed to indicate slight publication bias. However, Begg tests (P = 0.53) and Egger tests (P = 0.29) revealed no evidence to support publication bias in this pooled analysis. In addition, in the trim and fill method, none of the studied needed to be statistically corrected for funnel plot asymmetry.

## Discussion

PCOS has been repeatedly noted to be an independent risk factor of insulin resistance [46–50]. Thus, metformin, as the longest established oral antidiabetic agent, has been widely used in patients with PCOS to improve insulin sensitivity and induce ovulation. Adipocytokines, which are secreted by adipose tissue, may play a significant role in the pathogenesis of insulin resistance [51]. However, the effect of metformin on adipocytokine levels remains controversial. Some studies reported positive results [27–30, 34–40], whereas others found no significant changes in adipocytokine levels after metformin administration [26, 31].

The aim of the present study was to systematically review the literature for randomized, controlled, self-controlled trials that studied changes in the levels of adipocytokines (adiponectin, resistin, visfatin, and leptin) in patients with PCOS treated with metformin and to meta-analyze the best evidence available in order to provide high-quality data on the effect of metformin on adipocytokine levels in patients with PCOS. For this study, 34 data sets were used, including 744 patients with PCOS and adipocytokine levels measured before and after metformin administration. Thus, according to these results, metformin treatment in the patients with PCOS was associated with a significant increase in adiponectin level and decrease in leptin level, but not with the resistin and visfatin levels.

Similar to the findings of our meta-analysis, increased adiponectin levels have previously been observed in patients with PCOS who were receiving 850-mg metformin twice daily for 6 months [42, 43]. Insulin and glucose levels induced by activation of the AMP-kinase pathways have been shown to directly affect adiponectin levels [52, 53]. Increased adiponectin levels observed in patients with PCOS after metformin administration may be the result of reduced insulin resistance and insulin levels. However, inconsistent with our results, the results of another study [31] indicated no change in adiponectin concentration after treatment with 1000 mg of metformin daily for 6 months. These conflicting results could be explained by the relatively small sample size and difference in study populations, including differences in race, BMI, and severity of insulin resistance. Because of the complexity of the factors that influence the effect of metformin on adiponectin levels, we could not identify through our meta-analysis the precise dose or duration of metformin therapy needed to maximize the increase in adiponectin concentration in patients with PCOS. Nevertheless, based on the limited data available, our meta-analysis revealed that metformin treatment was associated with elevated serum adiponectin levels.

The role of leptin, as a marker of overall fat depots, in patients with PCOS is not well understood [54, 55]. Most studies reported higher leptin concentrations in patients with PCOS [56, 57], whereas others did not [58]. Various confounding factors (e.g., age and body weight) could have led to these conflicting results. Assessing the effects of the disease-specific treatment (metformin in this study) may be a way to address this issue. Based on the decreasing leptin concentrations after metformin administration in the patients with PCOS, our data seem to support the occurrence of hyperlipidemia, which is a state of leptin resistance or tolerance, in the patients with PCOS. Furthermore, leptin has been demonstrated to be associated with the development of insulin resistance and diabetes [17]. The result of our meta-analysis suggested that metformin administration was significantly associated with the reduction in circulation leptin concentration and that delaying or preventing the occurrence of insulin resistance and diabetes might be beneficial in patients with PCOS.

In addition, we found no significant changes in resistin or visfatin level during metformin treatment in the present study. A recent meta-analysis revealed no statistically significant differences in serum resistin and visfatin levels between patients with PCOS and control women, although serum resistin and visfatin levels were higher among both obese and normal-weight patients with PCOS than in the controls [59]. No correlation was observed between resistin level, BMI, homeostasis model assessment-estimated insulin resistance, area under the curve of blood insulin, insulin, lipid parameters, and serum androgen levels [60]. Meanwhile, Pagano and colleagues reported that insulin resistance did not lead to changes in the circulating plasma visfatin concentrations in human subjects [61]. These results might suggest that both resistin and visfatin levels were independently associated with insulin resistance in patients with PCOS.

Considering that more than half of the patients with PCOS had central obesity and many investigators found that metformin treatment induced a significant reduction in BMI [62–64], we performed a special meta-analysis regarding the effect of metformin administration on BMI in patients with PCOS for each adipocytokine. We found that metformin therapy could decrease BMI and blood concentrations of adiponectin and leptin. Moreover, we found no significant changes in BMI in the women with PCOS whose resistin and visfatin levels were unchanged. Thus, this may imply a significant correlation between the both adiponectin and leptin levels and BMI in patients with PCOS. Such results were also reported by many other authors. Orio’s and Panidis’s groups demonstrated a negative correlation between plasma adiponectin level and BMI in patients with PCOS [65, 66]. Similarly, the authors reported a positive correlation between plasma leptin level and BMI [67]. Nevertheless, the reasons for the conflicting BMI results are not clear. A possible explanation is the limited numbers and sample sizes of the studies included in the analysis of the effect of metformin on BMI in terms of resistin and visfatin levels. Meanwhile, another study reported that only about 50% of women with PCOS responded to metformin [68].

Although our findings are important, the limitations of our study should not be ignored. Presence of confounding variables (e.g., age, BMI, and concomitant subclinical inflammatory diseases), lack of uniformity (e.g., PCOS diagnostic criteria and assay methodology for index markers), and exclusion of studies not written in English are limitations that are difficult to avoid. Moreover, various PCOS phenotypes could not be considered. Furthermore, more studies with a large sample size would allow for precise estimation of the effect, whereas the limited numbers and sample sizes of the studies included in this analysis led to a high degree of heterogeneity in the reported resistin and visfatin levels, increasing the difficulty of interpreting the results and limiting the strength of the evidence provided by the present meta-analysis. The observed lack of effect should be interpreted as a failure to document any potential existing effect of metformin on resistin and visfatin levels in patients with PCOS rather than as evidence of no effect [69].

## Conclusion

The present meta-analysis demonstrates that metformin administration was associated with increased serum adiponectin concentration and decreased serum leptin levels. Further study is needed to elucidate whether this apparent effect decreases the incidence of type 2 diabetes and other metabolic diseases in patients with PCOS later in life.

## Supporting Information

S1 Table PRISMA checklist. (DOC)
